# The Cyclopædia of Practical Surgery

**Published:** 1842-01-01

**Authors:** 


The Cyclopædia of Practical Surgery, embracing a complete View of all the Departments in Operative Medicine. Edited by *William B. Costello*, M.D. Member of several learned Societies National and Foreign. Part X. London, Sherwood and Co. 1841.

The Part of the Cyclopædia before us is certainly not inferior to its predecessors, perhaps rather the reverse. The later Parts have, in our opinion, improved in style as well as matter. The former has been purged

---

of much tendency to Gallicisms that disfigured it—and the latter has acquired more of a practical character. We trust that in both respects the improvement will be progressive.

### ENCEPHALOCELE. By T. SPENCER WELLS, R.N.

Encephalocele, or hernia cerebri, by which term it is better known with us, is fairly handled by Mr. Wells. After observing that it is divided into accidental, and congenital or spontaneous, he restricts his observations to the latter.

If the sutures or fontanelles are larger or less firm than usual, from slow or defective ossification—if those which are naturally early consolidated continue fibrous longer than usual, or are separated by cerebral effusion—if there be a deficiency in some part of one of the bones of the head, or a fissure have occurred in labour, the walls of the cranium are incapable of resisting the pressure exercised upon them by the brain; or, these being normal, if the brain be unduly developed, wholly or in part, protrusion almost necessarily follows—the weaker parts yielding to the unceasing pressure.

This loss of balance in some cases, will depend on mechanical influences, to which the fœtus is exposed during its intra-uterine life—in others, on some more occult aberration from the normal process of growth and nutrition. Spasmodic uterine contractions would act in compressing the head, if the liquor amnii were deficient, and this may be occasionally the initial cause; but rarely, for the liquor amnii is in far greater proportion, with regard to the bulk of the fœtus, in the early months, than afterwards, when the structures are less delicate and require less protection. That a morbid growth from the uterus may produce changes, not only in one, but in several children, exposed to the same cause, is proved by a case referred to by Frank, of a woman who bore four children, each of whom had a deep depression on the forehead, the bone beneath which was not ossified. This woman had a pointed bony tumour in the back part of her pelvis. Blows received by the mother during pregnancy will occasionally produce the same effect. Roux gives a case of hernia of the posterior part of the encephalon in an infant, whose mother fell from a considerable height in the seventh month of pregnancy.

Faulty position of the child may check the progress of ossification. Thus Cruveilhier, in his remarks on a case in which the posterior part of the occipital bone was absent, ascribes it to a *renversement de la tête en arrière* during gestation. Otto says, that, in all the cases he has observed, the hernia was owing to effusion; and the defective ossification of the cranial bones was one of the consequences of the effusion.

When no mechanical cause can be traced, encephalocele must either be attributed to the primitive germ having been incomplete or defective; or to accidental malformation, resulting from unknown causes, which impede the regular progress of organization—which, or how, it is difficult to say. Mr. Wells seems to deny that encephalocele can be occasioned by compression of the head in its passage through the pelvis during labour.

*Seats of Encephalocele.*—Mr. Wells presents us with a Table of the seat of the protrusion in sixty-three recorded cases.

| | |
|---|---|
| Through an opening in the occipital bone | 24 |
| the foramen magnum | 7 |
| posterior fontanelle | 4 |
| anterior ditto | 3 |
| At the root of the nose | 5 |
| Between os unguis and os frontis | 1 |
| Lambdoid suture | 2 |
| Temporo-occipital suture | 1 |
| Fronto-parietal suture | 1 |
| Frontal suture | 8 |
| Between frontal and nasal bones | 2 |
| Just over right eye | 2 |
| left eye | 1 |
| Deficiency of temporal bones | 1 |
| Through base of skull | 1 |
| | 63 |

The coverings are the skin and subcutaneous cellular tissue, epicranial aponeurosis, dura mater and arachnoid. Delpech enumerates the pericranium as one of the coverings, and Breschet describes it in his fourteenth observation. All these layers however are so united that they cannot be separated.

The size ranges from that of a nut to a magnitude even exceeding that of a child's head. The vertebral canal is often more or less modified; the spinal column bifid, sometimes in the neck, back, or loins, more rarely throughout its whole extent. The medulla spinalis may be unaltered, but it is often atrophied throughout, and covered with layers of false membrane placed in the subarachnoid tissue. In some cases it has been replaced by a reddish cellular tissue infiltrated with a serous fluid. It is frequently soft and injected.

*Diagnosis.*—The diseases for which encephalocele has been mistaken, are cephalhæmatoma, various tumors of the scalp, nævus, fungus of the dura mater, serous cysts of the head, hydrocephalic protrusion, and nasal polypus.

The tumors of the scalp which may resemble encephalocele, are the bloody tumor, or sero-sanguineous œdema, so common on the heads of infants after protracted labour or instrumental delivery; and certain encysted or non-encysted tumors of the skin, or subcutaneous cellular tissue, the former class including the species formed by the retention of sebaceous matter in a dilated follicle, meliceris, and atheroma; the latter lipoma, and steatoma.

The swelling caused by infiltration of bloody serum, or extravasation of blood into the sub-cutaneous, or sub-aponeurotic cellular tissue of the scalp, can only resemble encephalocele when the effused matter has become surrounded by a hard ridge, resulting from the effusion of lymph, and consequent condensation of the parts immediately around it, which sometimes occurs, leaving the central part comparatively soft and yielding. In no other case is there any thing resembling an aperture in the walls of the cranium; and this ridge cannot be mistaken for the edge of a bony opening, if the finger be pressed steadily on it; for under continued pressure it will gradually disappear, whereas a bony edge would become more evident. And further, these swellings are unattended with pulsation, they are not diminished on pressure, nor are cerebral symptoms produced thereby.

The encysted and non-encysted tumors are distinguished by their superficial situation, indolent nature, and their mobility. They do not adhere to the bones, and the latter can be felt perfect beneath them. They also want the pulsation and other marks of encephalocele.

Nævi resemble encephalocele in pulsating, and being reduced in size by pressure; but their superficial situation, and want of communication with the cranium, are sufficient to distinguish them.

Fungus of the dura mater, in common with encephalocele, is partly reducible on compression, and loss of sensibility or coma are also produced. It is the seat of pulsation, and of a movement corresponding with inspiration and expiration. It passes through an opening in the bone. The common characters being so numerous, perhaps the marks of diagnosis will be best given by means of a table.

| ENCEPHALOCELE. | FUNGUS OF THE DURA MATER. |
| --- | --- |
| No premonitory symptoms. | Preceded by cephalalgia, and lancinating pains during the absorption of the bone. |
| Is congenital, or appears before ossification has been completed. | Excessively rare in infancy, but comes on at a period when the bones, sutures, and fontanelles, are completely consolidated. |
| Situated either on a suture, fontanelle, or some part of the skull, membranous during intra-uterine life. | Most frequently pierces the substance of a bone. |
| Borders of opening smooth. Only one exception to this rule on record. | Borders of opening rough and irregular. |
| Frequently pediculated. | Always large at base—never pediculated. |

A protrusion of the dura mater filled with fluid would not be readily mistaken for the soft, doughy, elastic tumor or simple encephalocele; but it might be impossible to distinguish the former from a hydrencephalocele; for in both cases the cranium is perforated, the dura mater forms the hernial sac, and the serum contained in the sac passes into the cranium on pressure being applied to the tumor, and compresses the brain. The diagnosis then might be impossible: but it is unimportant, as the treatment would be the same in both cases.

Dupuytren was once called to remove what was supposed to be a nasal polypus, but discovered it to be a cerebral protrusion; and another case of encephalocele, which had passed through an opening in the ethmoid bone into the nasal fossa, was mistaken for a polypus, and operated on by Richter.

*Prognosis.*—When the hernial tumor contains a very large portion of brain, death alone can be expected. When the tumor is small, the patient *may* reach adult age, though that is far from probable.

*Treatment.*—The leading plans have been ligature—incision or excision —puncture—compression.

A. *Ligature.*—There have been *three* recorded cases and three deaths. It is to be hoped there will be no more of either.

B. *Incision and Excision.*—Mr. Wells has collected the particulars of nine cases in which this disease has been treated by incision or excision, in seven of which the operation was followed by death. The most speedily successful case is that, related by Held, of a woman, nineteen years of age, who had a tumor the size of a rennet-apple to the left of the frontal suture. It yielded a little on pressure, and appeared to contain a fluid. He took it for meliceris, and determined to remove it; but, after incising the skin, he recognised an opening in the cranium below the fontanelle, and found that the tumor passed through this opening. It was covered by a membrane, which appeared to contain liquid. He incised this membrane, when two ounces of a yellowish fluid escaped, and the tumor collapsed; and he perceived pulsation and a portion of brain. Dry dressing was immediately applied, and gentle pressure employed, until the brain was reduced. The opening afterwards completely closed. The woman perfectly recovered, and afterwards had several children. Mr. Wells remarks, that the compression employed would, in all probability, have been equally beneficial if employed alone, and certainly, if conjoined with puncture. Incision appears to be equally unnecessary, useless, and dangerous.

C. *Puncture.*—Mr. Wells relates the particulars of six cases in which this was performed. Of these patients four died; in one the result was not ascertained; and Mr. Adams's single case is the only instance of cure —and this, it will be seen, was imperfect. It would appear, therefore, that though puncture may be less dangerous than incision, it is by no means free from risk; for it is extremely probable that many of the patients whose cases have just been detailed, would have lived much longer if the disease had been left to nature. The successful case of Mr. Adams' is the following:—

Puncture was performed seven times with a needle, and once with a lancet; and on this occasion alone did the operation *itself* seem to be followed with any fever or unusual restlessness in the infant. Pressure was once applied by means of plaster and bandage; but convulsions came on, and the pressure was never repeated. After repeated punctures, the quantity of fluid became so trifling that the operation was no longer necessary; but the solid part of the tumor, formed of the brain itself, was not diminished. The treatment "kept the disease from progressing until the child arrived at that state of development when the brain and its membranes became less disposed to watery secretions, and the powers of the constitution enabled the infant to provide a stronger skin, capable of sustaining the weight of the hernia.

Mr. Wells sums up:—The only circumstance under which our present experience can lead us to expect benefit from puncture, are probably when the sac is so much distended that its rupture or ulceration is almost certain. This occurrence is proved to have been fatal in so many instances, that it would be far better to anticipate it by puncture; thereby evacuating the fluid more gradually, and rendering the healing of the opening in the sac far more probable. This result is facilitated by making the punctures in those parts of the sac least affected by the effects of distention.

Mr. Wells has not been able to discover more than two successful recorded cases.

The first is the case of M. Salleneuve, which was related to the French Royal Academy of Surgery. The patient was a child, who was born with a tumor on the posterior and lateral part of the head,—soft; disappearing on compression; occupying the situation where the parietal, occipital, and temporal bones approximate each other; passing through an opening an inch and a half in diameter; and regularly pulsating. A piece of sheet-lead, pierced with holes, by which it could be fastened to the cap, was applied so as to make a moderate degree of permanent compression upon the tumor. It gradually diminished in volume, ossification proceeded, and the lamdoid suture was eventually perfectly closed. A case was also treated in a similar manner by Callisen. He does not give the details, but refers to it in the following terms: "Sic parvas hernias cerebri curari, ac hiatum osseum denique occludi posse, aliorum et *propria experientia* evicit." But he agrees, that when of a larger size, scarcely anything can be done beyond applying some contrivance to shield the protruded parts from injury.

Our author concludes—that when an encephalocele is not very large, and is reducible without producing evidence of cerebral compression, the displaced parts should be returned, and pressure applied by means of a plate of ivory, silver, or lead, maintained by a bandage. Ivory is preferable to a metallic support, as it is not so good a conductor of caloric, and therefore less capable of transmitting variations of temperature to the brain. If reduction be impossible,—and pressure causes no pain or dulness,—compression may be still applicable to prevent further enlargement. Lastly, if the tumor cannot be returned, nor pressure supported, all we can do is to adopt some mechanical contrivance, varied according to the form and situation of the tumor, to protect the brain from external injury, and from cold. It may be advisable to combine puncture with compression under the circumstances before stated.

A well executed article.

### ENDOSTEITIS.

For an account of this, we are indebted to Professor Walshe. This gentleman has coined the word *Endosteum*, as representative of the medullary membrane of voice, and *Endosteitis* signifies its inflammation. To this, we suppose, there can be no further objection than lies to the multiplcation of names.

The author's account of the pathological condition of the inflamed membrane is succinct. Increased vascularity, he says, forms, as elsewhere, an important feature in the anatomical condition of the inflamed endosteum; the injection is commonly extremely minute, so much so, that in some instances the medulla appears of a uniform florid or livid red colour. It is not very unusual to find blood extravasated in points or patches, and small hæmatomata are sometimes seen. The stage of exudation takes place with extreme rapidity. The medulla and meshes of the endosteum become infiltrated with liquid fibrinous matter, which at first causes softening of the infiltrated tissue; it nevertheless possesses the plastic property in so high a degree, that it almost immediately becomes concrete, increases the dimensions and firmness of the medulla, and causes thickening of the membrane itself. This is best seen in cases of endosteitis following amputation, where the medulla protrudes in the form of a small rounded deep red coloured mass on the surface of the stump, to such extent sometimes, as to require removal with the knife. When, as may be the case, the surgeon is obliged to repeat this little operation, in consequence of re-production of the removed part, the protruding mass at length ceases altogether to contain adipose matter, and is wholly composed of solidified fibrine. The contents of the cells of the cancellated structure, if the inflammation spread to them, undergo similar fibrinous infiltration.

This stage may be brief, and that of suppuration succeed. Pus may accumulate in minute spots, which by increasing and running into each other form abscesses of some size (a size of course always proportional to the space in which suppuration occurs); or a greater or less extent of the tissue may be infiltrated with that fluid. The infiltration extends to the cancellated texture.

Sphacelus is a not unfrequent termination. Necrosis of the internal stratum of the shaft follows.

Endosteitis may be acute or chronic. Examples of chronic endosteitis are those cases of abscess of the central cavity, and more especially of the articular heads of the bones, of which not a few examples are now upon record. A rarer appearance in the chronic state is induration and thickening, with grey or dirty-red discolouration of the medulla, attended with shrivelling of its substance. This shrivelling of tissue manifestly depends upon the slow contraction, co-advancing with the organization of the fibrine exuded during the acute stages. And, in all probability, the grey discolouration (depending immediately upon impaired freedom of the circulatory movements) the induration and reduced size of the endosteum and medulla, all of them so clearly traceable to the contractile power of the fibrine, are not the most important phenomena due to it. Dr. Walshe conjectures that the contraction in question may so far obstruct the motion of the blood in the vessels as nearly or totally to arrest the circulation in the adjoining bony layers. Even the occurrence of rupture of the delicate communications between the membrane and the bone appears to be in this manner not impossible. Now if either of these states is produced, necrosis of the bony layers referred to must be the result. The correctness of this idea remains to be determined.

Chronic, even more commonly than acute, endosteitis leads to changes in the bony structure characteristic of inflammatory action. It plays a leading part in central or articular caries. Endosteitis, too, is among the causes of the purulent impregnation of the blood accompanied by metastatic abscesses in the viscera, the lungs and liver especially, which prove among the most frequent causes of death in operations requiring division of bony structure. In injuries of the head, this inflammation affecting the diploe, holds the same relation to the purulent collections in the liver. That these secondary purulent accumulations are the result of suppuration in the endosteum, is firmly established by the fact, that in some such cases no other tissue in the body is the seat of primary suppurative inflammation. Probably the veins of the membrane are implicated.

But the particular causes of endosteitis, and its distinctive symptoms, are not so clear as its pathological characters. Nor is it likely that we shall ever be much better able to distinguish in the living body an affection, which cannot long remain insulated, and must usually be, at the best, but the initiative of others. Fortunately, this inability is of little practical importance.

### Epistaxis.

In treating of this Mr. Spencer Wells, the author of the article, describes an apparatus of M. Martin St. Ange for plugging the nares.

It consists of a straight tube, four inches in length, widened into the form of a cone at the extremity, which is not to be engaged in the nose, and terminating at the other by a small perforated nipple. The widened extremity has two rings like a catheter, and a small cock at the distance of five lines. Beyond this a slide plays, which may be tightened at pleasure by a screw. For the extent of an inch from the other extremity, circular grooves are made, and a small bladder, formed of the cœcum of a sheep, is fixed on the grooved extremity by a firm ligature. To be still more sure that the bladder may not be thrown off from the tube, it is connected by a thread with one of the rings at the handle. The bladder, being softened and folded around the tube, is introduced towards the pharynx, and filled with air or water by injection, which is retained by turning the cock. Slight traction is then employed to draw the small balloon closely against the posterior aperture of the nares. A piece of linen is placed in the orifice of the nares, on to which the screw is to be advanced, and the instrument fixed by its pressure. The whole apparatus can be withdrawn at will by opening the cock, when the bladder, more or less empty, brings forwards the clots contained in the nose.

We should apprehend that, to be efficient, this instrument would make inconvenient pressure on the anterior aperture of the nares. This perhaps might be obviated by an oval plate applied against the aperture and embracing the instrument. But the common means of plugging usually answer very well, and tools ought not to be unnecessarily introduced into the surgical chest.

### Erysipelas

Is a subject which will always interest both the surgeon and physician. It is treated by Mr. Donellan. We shall advert to some parts of the article.

*Influence of Seasons.*—There sometimes exists at certain seasons, and even during a course of years, a peculiar condition of the atmosphere, wholly inappreciable by any physical means of investigation, which disposes so completely to erysipelas, that the slightest exciting cause, the smallest abrasion or most superficial excitation of the skin, almost infallibly determines the development of the affection. Sometimes this influence is such that there is no need of even an exciting cause, and the disease assumes a real epidemic character. It is, then, impossible not to admit the existence of an erysipelatous constitution of the atmosphere. Tozzi speaks of an aggravated form of epidemic erysipelas that prevailed at Naples during the Autumn and Winter of the year 1700. A similar epidemic raged at Toulouse in 1716, which, from the mortality it caused, was compared to the plague. Bromfield mentions an epidemic erysipelas of the head that lasted two years, in which the antiphlogistic treatment was generally attended with fatal results, and bark and cordials were most serviceable. During the epidemic that prevailed in Paris in the year 1828, Dupuytren was reduced to the necessity of postponing all operations that were not urgent to perform, and in the lunatic asylum at Charenton all external revulsions, which constitute the basis of the treatment of mental alienation, were obliged to be suspended.

*Contagiousness?*—Mr. Donellan would almost seem to think erysipelas contagious in England but not in France. A whimsical notion. We fancy that it is contagious in all parts of the world when the circumstances favouring contagion are present, and not contagious when they are absent. If the person who is *exposed* is *predisposed* and there are concentrated effluvia and imperfect ventilation, we apprehend that there would soon be evidence of contagion on either side of the channel.

*Superior liability of the Female Sex?*—Women are more subject to it than men; but not, however, in the proportion of four to one, as Frank found it in the Institute of Pavia, when out of twenty erysipelatous patients sixteen were women. Out of twenty patients affected with erysipelas received into Professor Chomel's clinical wards at *La Charité*, thirteen were women. In 630 cases of erysipelas, distributed by the *Bureau Central* to the various hospitals of Paris during the years 1830 and 1831, there were 326 females. In 43 cases of erysipelas observed by Louis, 25 were women.

*Precursory Affection of the Lymphatic Glands.*—Though general premonitory symptoms usually usher in erysipelas, sometimes there are none to arrest attention. At others, a painful tumefaction of the lymphatic ganglia appears. This tumefaction always manifests itself in the ganglia appertaining to the parts upon which the eruption is to take place, in the cervical ganglia under the jaw or behind the ears, in erysipelas of the face and scalp, and in the axillary or inguinal, ganglia in the erysipelas of their respective extremities. It is in erysipelas of the face that this circumstance is most frequent and significant. Mr. Donellan has often seen M. Chomel foretel the impending erysipelas of this region before either redness, pain, or tension existed. There is hardly any risk of having our prediction belied when shiverings, followed by fever, are accompanied with pain and swelling of the subcutaneous lymphatic ganglia under the jaw, provided there is no angina or other affection to explain the local and general symptoms.

*Sudden Disappearance of Erysipelas.*—M. Louis denies the possibility of the *delitescence* (that is the fashionable phrase) of erysipelas. He thinks that those authors who admit it, have confounded simple erythematous redness with erysipelas, in which he says the skin is not only red but has become hard, thickened, and evidently altered in its texture. But Mr. Donellan disputes M. Louis' position, and tells us that we read in a letter from Dr. Chrestien of Montpellier a case of erysipelas of the leg disappearing and followed by delirium, which was subdued by recalling the eruption to its primitive seat by the application of a blister. A similar case is recorded by M. Blandin; a traumatic erysipelas of the lower extremity treated by refrigerants was suppressed, and grievous cerebral symptoms ensued, which however promptly subsided on the re-appearance of the eruption round the wound subsequent to the use of mercurial frictions. It would certainly be bold to affirm that erysipelas does not suddenly disappear, but it may be safely said that it rarely does so. There are few of our readers, we dare say, who have seen it do so.

*M. Blandin's Notions of Simple and Traumatic Erysipelas.*—According to Blandin, lymphitis precedes and always predominates over the inflammation of the cuticle, and it is to this circumstance that he refers the greater gravity of traumatic erysipelas. In external erysipelas, he says, the morbific principle at first disturbs the whole economy, but it is soon thrown outward, localized, and exhausted, whereas in external erysipelas the lymphatic vessels distribute to the whole economy the fluids that have been vitiated by the local phlegmasia, thus continually furnishing toxic materials that are invading the entire organism.

Mr. Donellan appears to be fond of nosological divisions, for he enumerates and describes no less than *sixteen* varieties of erysipelas—either too many or too few—too many if only leading and essential characters are kept in view—too few if it is intended to describe every shade of the disorder. However, here is *his* synopsis of the varieties:—

*Apyretic Erysipelas.*
*Simple Erysipelatous Fever.*
*Inflammatory Erysipelas.*
*Bilious Erysipelas.*
*Adynamic Erysipelas.*
*Ataxic Erysipelas.*
*Œdematous Erysipelas.*

*Phlegmonous Erysipelas.*
*Traumatic Erysipelas.*
*Erysipelas of the Head.*
*Erysipelas of the Extremities.*
*Erysipelas of the Trunk.*
*Erratic Erysipelas.*
*Periodic Erysipelas.*
*Infantile Erysipelas.*
*Senile Erysipelas.*

*Nature of Erysipelas.*—The following somewhat grandiloquent language presents us with Mr. Donellan's ideas on this point.

"If we consider erysipelas solely with reference to its local manifestations, it is impossible not to recognize in it the fundamental characters, if of not a frank and legitimate inflammation, at least of a fluxionary movement, with modifications varying according to the nature of the causes that have given development to it; but if we consider it in relation with the entire organism, and this is the proper view to be taken, we find its scope enlarged and its special genius declare itself, and it would be highly erroneous to see in it a pure inflammation, a disease circumscribed in the limits of its topical manifestations: an error that would be highly injurious in its results, as it would lead to therapeutical indications that would be insufficient or hurtful in their application."

The plain English of this is, that erysipelas partakes of the inflammatory and exanthematous characters.

*Mercurial Ointment for Erysipelas.*—Our readers are aware that this application has been used and given up and used again. The last advocate of its employment is M. Ricord, who assures us, of course, that *his* method is *the* method. This being the case, it is worth knowing.

According, then, to Ricord, the whole of the erysipelatous surface, with a small edging of the healthy skin, should be lightly smeared over with *recently prepared* mercurial ointment; once a day is sufficient if the ointment be not rubbed off; but if this occurrence should take place, it will be necessary to repeat the incrustion. As soon as the swelling subsides and the epiderm wrinkles, although the redness and heat may still persist, the use of the ointment should be discontinued, as this circumstance is the signal of the diminution of the phenomena of exhalation, and of the tendency towards the establishing of the equilibrium between this function and the absorption. If, he adds, the mercurial applications be still persisted in, the absorption soon gets the ascendency, and accidents of mercurial absorption, salivation, diarrhœa, &c., may be determined. These inconveniences may also take place if the healthy skin be covered with the ointment, and without the advantage of preventing the extension of the erysipelas, as we see it act in several cases of syphilitic affections, covering the symptoms once produced but not preventing their development. The erysipelas is to be thus pursued on each new part it may extend to, unless there be no tumefaction, for in this case the ointment should not be applied or danger of salivation will be again incurred. It is also useless and injurious to apply the ointment on the phlyctænæ. To prevent salivation during the treatment, he recommends the use of an aluminous gargle, and should it notwithstanding supervene, it should be promptly suppressed by the application of hydrochloric acid to the gums.

*Bleeding or Bark in Erysipelas.*—Perhaps extravagance has seldom been carried farther than in the treatment of erysipelas—one sect being as furious for bark as the other is for bleeding. Mr. Donellan seems to us to lean too much towards the latter, which, in large towns at all events, and in delicate people, is replete with danger. Nor are we very partial to the ultra use of bark or stimuli. As usual, we apprehend that the truth lies in the mean.

We must confess that we do not admire Mr. Donellan's style. That it is not English we are sure ; and we are also sure that it is not French. It is, in fact, a tertium quid between the two ; possessing the elegancies and imbued with the genius of neither. This mongrel *patois* which has been latterly imported ought to be in every way discouraged. We would recommend Mr. Donellan to study our best writers, discard as much as possible all foreign terms and turns, and say what he has got to say in the plainest English and the simplest manner. His opinions will lose nothing of their value, and gain much in their expression.

Nor do we deem Mr. Donellan blameless in the *matter* of his article. He appears to know more of what is done in France than of what is going on in his own country. The references to foreign authors are as profuse as those to British ones are meagre. The paper might pass for being written by one Frenchman and translated into English by another.

FARCY, by M. Rayer, is a good contribution, and we are presented with its history in man and in the horse.

FISTULA, too, is carefully compiled and will repay perusal. On the whole, the Part is far from a bad one.

